# Exploring the Linkages Among Chronic Illness, Substance Use, and COVID-19 Infection in Adults Aged 50 Years and Older: Retrospective Cross-Sectional Analysis of National Representative Data

**DOI:** 10.2196/63024

**Published:** 2024-10-15

**Authors:** Suebsarn Ruksakulpiwat, Atsadaporn Niyomyart, Chontira Riangkam, Lalipat Phianhasin, Chitchanok Benjasirisan, Jon Adams

**Affiliations:** 1 Department of Medical Nursing, Faculty of Nursing, Mahidol University Bangkok Thailand; 2 Ramathibodi School of Nursing, Faculty of Medicine Ramathibodi Hospital, Mahidol University Bangkok Thailand; 3 School of Public Health, University of Technology Sydney Sydney Australia

**Keywords:** multiple chronic conditions, medical complexity, co-occurring conditions, substance use, COVID-19, SARS-CoV-2, older adults, gerontology, geriatrics

## Abstract

**Background:**

The co-occurrence of chronic illnesses and substance use presents complex challenges for health care systems. Understanding the interplay between these factors, compounded by the context of the COVID-19 pandemic, is essential for effective intervention strategies.

**Objective:**

This study aims to investigate the relationships among chronic illness, substance use, and COVID-19 infection in adults aged 50 years and older.

**Methods:**

Participants were 1196 adults aged 50 years and older. Descriptive statistics were used to describe demographic information. Logistic regressions and multiple regression analyses were used to determine associations between chronic illnesses, substance use, and COVID-19 infection. Mediation analysis was used to determine the effect of chronic illness mediators in the association between COVID-19 concerns and substance use.

**Results:**

The mean age was 68 (SD 10.3) years, with 58.6% (701/1196) being women. Adjusted analysis revealed that age and sex (women) significantly predicted a lower level of substance use (*P*<.05). However, marital status (separated or widowed) and chronic illness significantly predicted a higher level of substance use (*P*<.05). Furthermore, having dementia, arthritis, and high cholesterol significantly predicted a higher level of concern about the COVID-19 pandemic (*P*<.05). Logistic regression analysis indicated that individuals with hypertension (odds ratio [OR] 1.91, 95% CI 1.37-2.66; *P*<.001), lung disease (OR 2.42, 95% CI 1.23-4.75; *P*=.01), heart condition (OR 1.99, 95% CI 1.28-3.10; *P*=.002), stroke (OR 2.35, 95% CI 1.07-5.16; *P*=.03), and arthritis (OR 1.72, 95% CI 1.25-2.37; *P*=.001) were more likely to have their work affected by the COVID-19 pandemic. The mediation analysis showed a significant effect of COVID-19 concern on substance use through the mediation of chronic illness, with a 95% CI of –0.02 to –0.01 and an indirect effect of –0.01.

**Conclusions:**

Our study reveals complex associations among chronic illnesses, substance use, and COVID-19 infection among adults aged 50 years and older. It underscores the impact of demographics and specific chronic conditions on substance use behaviors and COVID-19 concerns. In addition, certain chronic illnesses were linked to heightened vulnerability in employment status during the pandemic. These findings emphasize the need for targeted interventions addressing physical health and substance use in this population during the COVID-19 pandemic.

## Introduction

### Background

Chronic illnesses are persistent health conditions requiring long-term management [1]. Common examples include diabetes [2], cardiovascular diseases [3], and respiratory infections [4], which contribute significantly to the increasing burden of chronic conditions among adults aged 50 years and older [5,6]. The high prevalence of these conditions highlights the importance of examining the factors that influence health trajectories in this age group [7]. While managing chronic illnesses is critical, it is also important to consider other health issues that can compound the challenges faced by this demographic. Among these, substance use problems stand out as a significant concern. Substance use, which includes excessive alcohol and drug consumption, not only jeopardizes mental and physical health but also complicates the management of chronic illnesses [8,9]. The relationship between chronic illnesses and substance use is intricate, with substance use potentially exacerbating chronic conditions and vice versa. A study using electronic health records from the United States found that 48.3% (102,324/211,880) of individuals were diagnosed with at least 1 chronic disease [10]. Furthermore, those with at least 1 substance use disorder had higher odds of having a chronic disease [10]. Another retrospective study explored the association between chronic disease and substance use among older adults and found that marijuana use and smoking were significantly associated with chronic disease, while alcohol use was not [11]. This interplay highlights the need for a comprehensive approach that addresses both chronic conditions and substance use, ensuring that health care strategies are well-rounded and effective in managing the multifaceted health needs of older adults.

As the COVID-19 pandemic continues to reshape the health care landscape, the interplay between chronic illnesses and substance use has grown increasingly complex. Beyond the direct impact of the virus, the pandemic has introduced additional stressors and challenges, influenced health behaviors, and exacerbated preexisting health conditions [12-14]. In particular, the pandemic has intensified the prevalence and severity of chronic illnesses, while also disrupting health care access and support systems. A study shows that the COVID-19 pandemic has worsened chronic illnesses by increasing their severity and risk. Patients with conditions such as cardiovascular diseases and diabetes experience more severe outcomes due to factors such as elevated angiotensin-converting enzyme 2 levels and cytokine storms. In addition, the COVID-19 pandemic can also induce new chronic conditions, highlighting the need for targeted management strategies [15]. The pandemic’s impact on substance use patterns is of particular interest. Factors such as isolation, economic uncertainties, and disruptions in health care access may contribute to shifts in how individuals cope with stress, potentially affecting substance use trends [16,17]. Amid this complex backdrop, the relationship between substance use and chronic illnesses takes on heightened significance [10]. While research has explored these factors individually, a comprehensive understanding of their interconnection—particularly within the unique context of COVID-19 pandemic—is lacking. It is plausible that both the effects of COVID-19 pandemic and chronic illness are related to substance use. Therefore, chronic illnesses could serve as a mediating variable in the relationship between COVID-19 infection and substance use. This study addresses this gap by conducting secondary data analysis on the HRS (Health and Retirement Study) 2020, a retrospective cohort study. The HRS 2020 represents American adults aged 50 years and older, investigating characteristics, health behaviors, and the unique context of the COVID-19 pandemic.

This research holds practical implications that extend beyond theoretical contributions. By identifying and elucidating the pathways through which chronic illness impacts substance use, our mediation analysis can inform public health strategies. This investigation aims to contribute to a comprehensive understanding of the multifaceted challenges faced by adults aged 50 years and older, ultimately enhancing the effectiveness of health care interventions and care for these vulnerable groups.

### Study Aims

**Figure 1:**
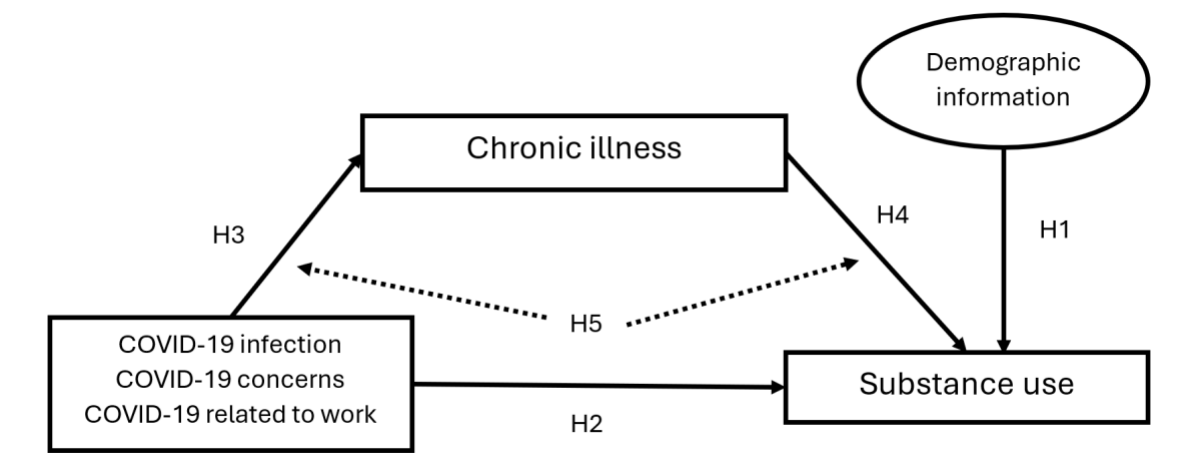
Hypothesis model. H: hypothesis.

This study aims to investigate the relationships among chronic illness, substance use, and COVID-19 infection in adults aged 50 years and older. We hypothesized that the undesirable demographic factors of adults aged 50 years and older (ie, lower number of years in school, being separated or divorced or widowed) would be positively associated with substance use (hypothesis 1). Furthermore, we anticipated that higher odds of COVID-19 components, including COVID-19 infection, COVID-19–related concerns, and its relationship to work (hereinafter “COVID-19 related to work”), would increase the likelihood of substance use (hypothesis 2). In addition, we hypothesized that the odds of all COVID-19 components would be associated with a number of chronic illnesses (hypothesis 3) and a higher number of chronic illnesses would be positively linked with the likelihood of substance use (hypothesis 4). Finally, we expected that the direct effect of all COVID-19 components would be mitigated and the indirect effect through chronic illness would be significant among adults aged 50 years and older, thus revealing a mediating role of chronic illness (hypothesis 5; Figure 1).

## Methods

### Study Design and Data Collection

The research was carried out following the STROBE (Strengthening the Reporting of Observational Studies in Epidemiology) guidelines for cross-sectional studies (Multimedia Appendix 1). In this retrospective cross-sectional study, we used public data from the HRS, an ongoing longitudinal panel survey [18]. The HRS sample follows a multistage, stratified study design conducted every 2 years since 1992, interviewing approximately 20,000 Americans aged 50 years and older [19]. This study used the HRS 2020 Final Release, HRS 2020 Tracker Final Release, and HRS 2020 Experimental Modules (specifically, Module 10: Substance Use Problems). In every wave, HRS incorporates numerous experimental modules (eg, Module 10: Substance Use Problems), which are conducted following the core interview. These modules, typically lasting 2-3 minutes, are deliberately crafted to address various topics, including both new subjects and those that augment the core information. While sample sizes may vary, they generally constitute approximately 10% of a random sample drawn from the core. Importantly, each respondent is assigned only 1 experimental module per wave.

**Figure 2:**
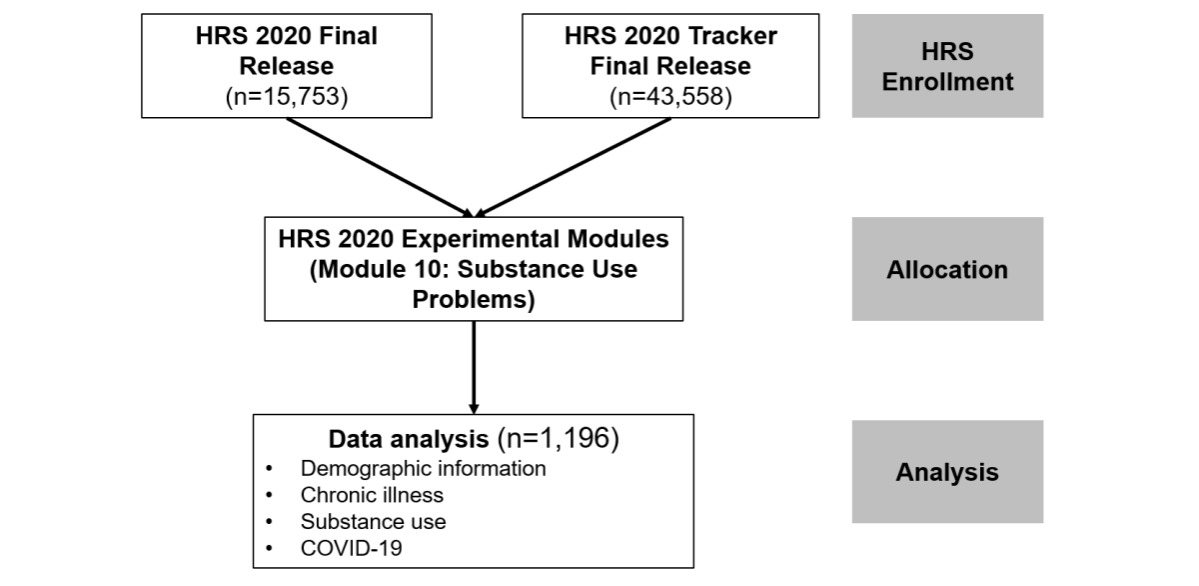
Study flowchart. HRS: Health and Retirement Study.

For this study, 15,753 participants were recruited from the HRS 2020 final release, and 43,558 people were included from the HRS 2020 Tracker final release. After including only those who participated in Module 10: Substance Use in Experimental Modules, 1196 participants were eligible and will be included in the final analysis (Figure 2).

### Measures

#### Demographic Information

In this study, demographic information included age (in years), sex (“men” or “women”), race or ethnicity (“White/Caucasian,” “Black/African American,” or “Other”), number of school years (“no formal education” [0 years], “grades” [1-11 years], “high school” [12 years], “some college” [13-15 years], “college graduate” [16 years], or “post college” [17 years or more]), and marital status (“married,” “separated or divorced,” “widowed,” or “never married”). Demographic data were extracted from the HRS 2020 Final Release and HRS 2020 Tracker Final Release to facilitate consistent use of HRS data across waves.

#### Chronic Illness

Chronic illnesses, as defined by the Centers for Disease Control and Prevention (CDC), are conditions that persist for 1 year or more and require ongoing medical attention or limit daily activities [1]. In this study, we examined 9 chronic conditions: hypertension, diabetes mellitus, lung disease, heart conditions, stroke, depression, dementia, arthritis, and high cholesterol. Participants were asked whether they had ever been diagnosed with each condition, with responses coded as binary: “No” was coded as 0, and “Yes” as 1. The total number of chronic conditions was summed to create a “Chronic Illness” variable, ranging from 0 to 9, where a higher score indicates a greater number of chronic illnesses. Data were extracted from the HRS 2020 Final Release, and recoding was performed as necessary to facilitate analysis.

#### COVID-19

COVID-19 variables consisted of 6 measures, which can be categorized into three categories: (1) COVID-19 Concern (assessed on a scale from 1 to 10, where 1 indicated the least concern and 10 the most); (2) COVID-19 Infection: Self-report (Had you had or did you then have COVID-19, the disease caused by the novel coronavirus?) and Test-based (Did any tests indicate that you had the virus?); and (3) COVID-19 Related to Work: Working Affected by COVID-19 (Was your work affected because of the coronavirus pandemic?) and Stop Working (Did you have to stop work entirely because of COVID-19?). The responses to the questions COVID-19 Self-report, COVID-19 Test-based, Working Affected by COVID-19, and Stop Working were “No = 0” and “Yes = 1.” All variables were extracted from the HRS 2020 Final Release. Original response categories for each variable were combined and recoded as necessary to facilitate data analysis.

#### Substance Use

Module 10 in HRS 2020 Experimental Modules [20] focused on issues related to substances, encompassing medications, other substances, tobacco, and alcohol that HRS participants might have encountered. Developed by the National Institutes of Health, these questions, now commonly posed in various medical settings such as doctors’ offices and hospitals, aimed to address concerns regarding substance use among Americans. The module prompted respondents to indicate how frequently they had used various substances over the past few months to a year.

In this study, we analyzed variables associated with the use of alcohol, tobacco, cannabis, prescription medications for nonmedical purposes, illegal substances, cocaine, stimulants, methamphetamines, inhalants, sleeping pills, hallucinogens, street opioids, and prescription opioids. Responses to these questions were coded as “Never = 1” and “At least 1 time (including once or twice, monthly, weekly, daily, or almost daily) = 0.” Substance use was then coded such that a higher total score indicated less frequent use, with scores ranging from 0 to 13, where 0 represented the most frequent use.

### Statistical Analysis

In this study, we combined the HRS 2020 Final Release, the HRS 2020 Tracker Final Release, and the HRS 2020 Experimental Modules’ respondent-level analytic weights and used the combined weight in the analyses. Listwise deletion, which means deleting cases with missing data, was used to deal with the missing data [21]. Analyses were conducted using SPSS Statistics (version 20.0; IBM Corp). In our study, demographic information of participants was assessed using descriptive statistics such as percentages or means as appropriate. Logistic regressions and multiple linear regression analysis were used for associations where the outcome variable was categorical (binary) and continuous, respectively. For logistic regression, odds ratio (OR), 95% CI, and *P* value (significance level<.05) were reported as appropriate. For multiple linear regression, *R*^2^, adjusted *R*^2^, unstandardized and standardized b, SE, and *P* value were reported. The mediation analysis, the “PROCESS” Procedure for the SPSS (version 3.3), written by Hayes [22], was used. A total of 5000 bootstraps were applied to estimate 95% CI and evaluate the significance of indirect effects. Strategies proposed by Preacher and Hayes [23] were adapted to evaluate the pathway, and 4 necessary steps were followed to assess mediation. Step 1: association between the predictor and outcome (path c = X →Y; total effect or the sum of the direct and indirect effects); step 2: association between the predictor and mediators (path a = X → M); step 3: association between mediators and outcome (path b = M → Y); and step 4: association between the predictor and outcome after controlling for mediators (path c’ = direct effect). The indirect effect was the coefficient of the “a” path multiplied by the coefficient of the “b” path, representing the effect of X on Y through M.

### Ethical Considerations

The University of Michigan’s institutional review board (IRB) approved the HRS survey. The HRS provides financial payments as tokens of appreciation to respondents for their participation in various aspects of the study, rather than as compensation. For all regular study components involving enrolled participants, these payments are typically issued via check, included in advance letters informing them of upcoming participation requests. The primary exception is for baseline interviews with new participants, for whom there is no prior information available to issue checks in advance. In these cases, compensation is provided in cash at the time of the interview. Since 2016, the payment schedule for research activities has been structured as follows: US $100 for the baseline core interview, US $80 for the core panel interview, US $20 for the leave-behind after the core, US $25 for mail surveys, and US $50 for in-home venous phlebotomy. Moreover, informed consent is not required in this study as the original consent was obtained in the original HRS study. The data used in this study have been publicly released and deidentified, and are available from the HRS website. Therefore, it did not require ethical approval (project no. MU-MOU-IRB-NS 2024/13.2901 exemption approved by the IRB, Faculty of Nursing, Mahidol University).

## Results

### Sample Characteristics

Table 1 shows the characteristics of adult and older adult participants. Overall, 1196 participants were included. The mean age was 68 (SD 10.3) years, and most of them were women (701/1196, 58.6%), White individuals (765/1196, 64%), had completed high school (363/1196, 30.4%), and were married (631/1196, 52.8%). In terms of chronic illnesses, the mean number of chronic illnesses was 2.54 (SD 1.6). Also, 64.2% (768/1196) of participants had hypertension, 27.6% (330/1196) had diabetes mellitus, 11.1% (133/1196) had lung disease, 24.2% (289/1196) had a heart condition, 8.2% (98/1196) had a stroke, 26.7% (319/1196) had depression, 1.7% (20/1196) had dementia, 38.7% (463/1196) had arthritis, and 29.2% (349/1196) had high cholesterol. Regarding COVID-19 infection, the average concern score about the virus was 7.89 (SD 2.7). Only 3.3% (39/1196) of respondents reported contracting COVID-19 infection, while 2.2% (26/1196) tested positive for the virus. Concerning employment, 20.5% (245/1196) of participants indicated that COVID-19 infection had an impact on their work, with 9.4% (113/1196) of individuals halting work entirely due to the pandemic. As for substance use, the mean score was 11.37 (SD 0.85), ranging from 0 to 13; a higher score reflects lower levels of substance use.

**Table 1:** Characteristics of participants (adults aged 50 years and older).

Characteristics of participants^a^			Values
Age (years), mean (SD; range)			68 (10.3; 50-99)
**Gender, n (%)**			
	Men		495 (41.4)
	Women		701 (58.6)
**Race and ethnicity, n (%)**			
	White		765 (64.0)
	Black or African American		279 (23.3)
	Other		152 (12.7)
**Number of years in school, n (%)**			
	No formal education (0)		18 (1.5)
	Grades (1-11)		195 (16.3)
	High school (12)		363 (30.4)
	Some colleges (13-15)		312 (26.1)
	College graduate (16)		171 (14.3)
	Postcollege (≥17)		137 (11.5)
**Marital status, n (%)**			
	Married		631 (52.8)
	Separated or divorced		270 (22.6)
	Widowed		191 (16)
	Never married		104 (8.7)
**Chronic illness**			
	Number of chronic illness^b^, mean (SD; range)		2.54 (1.6; 0-9)
	Hypertension, n (%)		768 (64.2)
	Diabetes mellitus, n (%)		330 (27.6)
	Lung’s disease^c^, n (%)		133 (11.1)
	Heart condition^d^, n (%)		289 (24.2)
	Stroke, n (%)		98 (8.2)
	Depression, n (%)		319 (26.7)
	Dementia, n (%)		20 (1.7)
	Arthritis, n (%)		463 (38.7)
	High cholesterol, n (%)		349 (29.2)
**COVID-19**			
	COVID-19 concern, mean (SD)		7.89 (2.7)
	**COVID-19 infection**		
		Self-report, n (%)	39 (3.3)
		Test-based, n (%)	26 (2.2)
	**COVID-19 related to work**		
		Working affected by COVID-19, n (%)	245 (20.5)
		Stop working, n (%)	113 (9.4)
**Substance use**			
	Substance use^e^, mean (SD; range)		11.37 (0.85; 0-13)

^a^Listwise deletion, which entails deleting cases with missing data, was used to handle missing data.

^b^The sum of chronic illnesses includes hypertension, diabetes mellitus, lung disease, heart condition, stroke, depression, dementia, arthritis, and high cholesterol (score range between 0 and 9; a higher score indicates a higher number of chronic illnesses).

^c^This encompasses chronic bronchitis or emphysema.

^d^This encompasses heart attack, coronary heart disease, angina, congestive heart failure, or other heart problems.

^e^Substance use (score range between 0 and 13; a higher score indicates a lower level of substance use, including drinking alcohol, tobacco use, cannabis use, prescription drugs for nonmedical reasons, illegal drugs, cocaine, stimulants, methamphetamines, inhalants, sleeping pills, hallucinogens, street opioids, and prescription opioids).

### Association Between the Characteristics of Adults Aged 50 Years and Older and Substance Use

The association between the characteristics of adults aged 50 years and older and substance use is shown in Table 2. In model 1, where characteristics of participants including age, sex, race or ethnicity, number of years in school, marital status, and chronic illnesses are independent variables, and substance use is the dependent variable, a significant equation was found (*F*_6,589_=13.55; *P*<.001), with an *R*^2^ of 0.12 and adjusted *R*^2^=0.11. The results show that age (*B*=0.03; *P*<.001) and being women (*B*=0.18; *P*=.007) significantly predict a lower level of substance use. However, marital status (separate or widow; *B*=–0.11; *P*=.001) and chronic illnesses (*B*=–0.11; *P*<.001) significantly predict a higher level of substance use. After excluding race or ethnicity and number of years in school in model 1 as they are insignificant, age, sex, marital status, and chronic illnesses remain significant predictors of substance use (model 2).

**Table 2:** The association between the characteristics of adults aged 50 years and older and substance use^a^.

Characteristics			Unstandardized coefficients				Standardized coefficients		*t* test (*df*)			*P* value^c^
			*B* ^b^	SE		*b*						
**Model 1 (n=596)**												
	Constant	9.84		0.329	—^d^			29.87 (6,589)		<.001		
	Age (years)	0.03		0.004	.29			7.07 (6,589)		*<.001*		
	Sex (reference group: women)	0.18		0.066	.11			2.69 (6,589)		*.007*		
	Race/ethnicity (reference group: White)	0.06		0.052	.05			1.21 (6,589)		.23		
	Number of years in school	–0.01		0.010	–.02			–0.51 (6,589)		.61		
	Marital status (reference group: married)	–0.11		0.034	–.13			–3.20 (6,589)		*.001*		
	Number of chronic illness^e^	–0.11		0.021	–.21			–5.24 (6,589)		*<.001*		
**Model 2** **(n=596)**												
	Constant	9.94		0.27				37.15 (4,591)		<.001		
	Age (years)	0.03		0.01	.27			6.93 (4,591)		*<.001*		
	Sex	0.17		0.07	.10			2.58 (4,591)		*.01*		
	Marital status	–0.10		0.03	–.12			–3.02 (4,591)		*.003*		
	Number of chronic illnesses^e^	–0.11		0.02	–.20			–5.18 (4,591)		*<.001*		

^a^Dependent variable: substance use (score range between 0 and 13; a higher score indicates a lower level of substance use, including drinking alcohol, tobacco use, cannabis use, prescription drugs for nonmedical reasons, illegal drugs, cocaine, stimulants, methamphetamines, inhalants, sleeping pills, hallucinogens, street opioids, and prescription opioids).

^b^*B*: unstandardized *b*.

^c^Italicized values are significant at *P*<.05.

^d^Not applicable.

^e^The sum of chronic illnesses includes hypertension, diabetes mellitus, lung disease, heart condition, stroke, depression, dementia, arthritis, and high cholesterol (score range between 0 and 9; a higher score indicates a higher number of chronic illnesses).

### The Association Between Chronic Illness in Adults Aged 50 Years and Older and COVID-19 Concern

Table 3 shows the association between chronic illnesses in adults aged 50 years and older and concern about the COVID-19 pandemic. After adjusting for age, sex, race or ethnicity, number of years in school, and marital status in model 1, a significant equation was found (*F*_14,861_=5.81; *P*<.001), with an *R*^2^ of 0.09 and adjusted *R*^2^=0.07. The results indicate that having dementia (*B*=1.60; *P*=.02), arthritis (*B*=0.69; *P*=.001), and high cholesterol (*B*=0.49; *P*=.02) significantly predict a higher level of concern about the COVID-19 pandemic. In model 2, adjusting for sex and race or ethnicity, dementia, arthritis, and high cholesterol remain significant predictors of COVID-19 concern with slightly changed significance values.

**Table 3:** The association between chronic illness of adults aged 50 years and older and concern about COVID-19 pandemic^a^.

Characteristics		Unstandardized coefficients		Standardized coefficients		*t* test (*df*)		*P* value^c^
		*B* ^b^	SE	*b*				
**Model 1 (n=876)**								
	Constant	5.08	0.85	—^d^	5.97 (14,861)		<.001	
	Hypertension	0.28	0.20	.05	1.41 (14,861)		.16	
	Diabetes mellitus	0.21	0.21	.03	0.99 (14,861)		.32	
	Lung’s disease	–0.20	0.30	–.02	–0.66 (14,861)		.51	
	Heart condition	0.32	0.22	.05	1.44 (14,861)		.15	
	Stroke	–0.56	0.34	–.06	–1.65 (14,861)		.10	
	Depression	–0.14	0.21	–.02	–0.68 (14,861)		.50	
	Dementia	1.60	0.66	.08	2.44 (14,861)		*.02*	
	Arthritis	0.69	0.20	.12	3.49 (14,861)		*.001*	
	High cholesterol	0.49	0.20	.08	2.39 (14,861)		*.02*	
**Model 2 (n=876)**								
	Constant	5.10	0.39		13.15 (11,864)		<.001	
	Hypertension	0.28	0.19	.05	1.42 (11,864)		.15	
	Diabetes mellitus	0.21	0.21	.03	0.99 (11,864)		.32	
	Lung’s disease	–0.18	0.30	–.02	–0.62 (11,864)		.54	
	Heart condition	0.30	0.22	.05	1.40 (11,864)		.16	
	Stroke	–0.54	0.34	–.05	–1.62 (11,864)		.11	
	Depression	–0.12	0.21	–.02	–0.58 (11,864)		.56	
	Dementia	1.62	0.66	.08	2.47 (11,864)		*.01*	
	Arthritis	0.68	0.19	.12	3.58 (11,864)		*<.001*	
	High cholesterol	0.48	0.20	.08	2.39 (11,864)		*.02*	

^a^Dependent variable: COVID-19 concern (overall, on a scale from 1 to 10, where 1 is the least concerned and 10 is the most concerned, how concerned are you about the coronavirus pandemic?). Model 1: adjust for age, sex, race or ethnicity, number of years in school, and marital status. Model 2: adjust for sex and race or ethnicity.

^b^*B*: unstandardized *b*.

^c^Italicized values are significant at *P*<.05.

^d^Not applicable.

### The Adjusted OR of COVID-19 Infection and COVID-19 Related to Work in Adults Aged 50 Years and Older With Chronic Illness

The adjusted (sex and race or ethnicity) association between chronic illnesses and various COVID-19 components, including COVID-19 infection and COVID-19 related to work, was determined (Table 4). In model 1, the independent variables are chronic illnesses, and the outcome variable is work affected by COVID-19 pandemic (Was your work affected because of the coronavirus pandemic?). The results indicate that for individuals with hypertension (OR 1.91, 95% CI 1.37-2.66; *P*<.001), lung disease (OR 2.42, 95% CI 1.23-4.75; *P*=.01), heart condition (OR 1.99, 95% CI 1.28-3.10; *P*=.002), stroke (OR 2.35, 95% CI 1.07-5.16; *P*=.03), and arthritis (OR 1.72, 95% CI 1.25-2.37; *P*=.001), their work is more likely to be affected because of the COVID-19 pandemic. Moreover, model 2 shows the association between chronic illnesses and stopping work as an outcome (Did you have to stop working entirely because of COVID-19?). The results reveal that individuals with diabetes mellitus are 49% as likely to stop working entirely because of the COVID-19 pandemic compared with those with no diabetes mellitus (OR 0.49, 95% CI 0.25-0.95; *P*=.04). No significance was found in the model in which COVID-19 infections (both self-report and test-based) are dependent outcome variables, so they are not shown in Table 4 but can be found in Multimedia Appendix 2.

**Table 4:** The association between chronic illness of adults aged 50 years and older and COVID-19 infection and COVID-19 related to work^a^.

Chronic illness	COVID-19–related factors					
	Model 1^b^: working affected by COVID-19^c^ (n=876)			Model 2^b^: stop working^d^ (n=245)		
	*P* value	OR^e^	95% CI	*P* value	OR	95% CI
Hypertension	*<.001*	*1.91*	*1.37-2.66*	.48	0.82	0.47-1.44
Diabetes mellitus	.20	1.29	0.87-1.89	*.04*	*0.49*	*0.25-0.95*
Lung disease	*.01*	*2.42*	*1.23-4.75*	.23	0.43	0.11-1.68
Heart condition	*.002*	*1.99*	*1.28-3.10*	.67	1.21	0.52-2.81
Stroke	*.03*	*2.35*	*1.07-5.16*	.36	2.29	0.40-13.26
Depression	.50	0.88	0.60-1.20	.51	1.24	0.66-2.37
Dementia	.73	1.26	0.33-4.86	.45	0.33	0.02-5.77
Arthritis	*.001*	*1.72*	*1.25-2.37*	.95	0.98	0.57-1.71
High cholesterol	.93	0.99	0.68-1.42	.66	1.15	0.62-2.14

^a^No significance was found in the model in which COVID-19 self-report and COVID-19 test-based are dependent outcome variables; therefore, it was not shown in Table 4. The reference group consists of the respondents who did not report having any chronic illnesses. *P* values in italics indicate statistically significant results (*P*<.05).

^b^Adjusted for sex and race or ethnicity.

^c^Working affected by COVID-19: Was your work affected because of the coronavirus pandemic?

^d^Stop working: Did you have to stop working entirely because of COVID-19?

^e^OR: odds ratio.

### Examining the Role of Chronic Illness as a Mediator in the Relationship Between COVID-19 Concern and Substance Use

The effect of COVID-19 concern (X) on substance use (Y) through the mediator of chronic illness (M) was examined. The path from COVID-19 concern to chronic illness exhibited a significant positive relationship (*b*=0.12, SE=0.03; *P*<.001).

**Figure 3:**
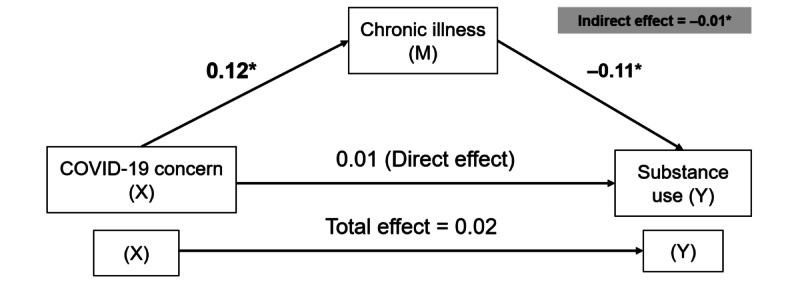
The mediation analysis model of the effect of chronic illness mediators (M) in the association between COVID-19 concern (X) and substance use (Y). *Statistically significant at *P*<.05.

The path from the chronic illness mediator to substance use exhibited a significant negative relationship (*b*=–0.11, SE=0.02; *P*<.001), suggesting that individuals with higher numbers of chronic illnesses were more likely to demonstrate a higher level of substance use. The direct effect of COVID-19 concern on substance use was positive and insignificant (*b*=0.01, SE=0.02; *P*=.57). The indirect effect was assessed using nonparametric 5000 bootstrapping. The 95% CI for the indirect effect (–0.01) was –0.02 to –0.01, indicating a significant effect of COVID-19 concern on substance use through the mediation of chronic illness (Table 5 and Figure 3).

**Table 5:** The effect of chronic illness mediators (M) in the association between COVID-19 concern (X) and substance use (Y).^a^

	COVID-19 concern (X) (n=460)													
Chronic illness (M)	X→M (a)				M→Y (b)				X→Y (c’)				Indirect effect (a×b)	
	a	SE	*P* value	*b*		SE	*P* value	c’		SE	*P* value	a×b		95% CI
	0.12	0.03	<.001	–0.11		0.02	<.001	0.01		0.02	.57	–0.01		–0.02 to –0.01

^a^Dependent variable: substance use (score range between 0 and 13; a higher score indicates a lower level of substance use, including drinking alcohol, tobacco use, cannabis use, prescription drugs for nonmedical reasons, illegal drugs, cocaine, stimulants, methamphetamines, inhalants, sleeping pills, hallucinogens, street opioids, and prescription opioids). Total effect (X→Y): effect 0.02 (95% CI –0.03 to 0.03).

## Discussion

### Characteristics of Adults Aged 50 Years and Older and Substance Use

Focusing upon adults aged 50 years and older, prior studies revealed that substance use is increasing in comparison with younger adults, and this trend has been well documented [24,25]. In our research, when considering characteristics of substance use in this demographic, we found that age, sex, race or ethnicity, number of years in school, marital status, and chronic illnesses explained 12.0% of substance use behaviors. However, only age, sex, marital status (being widowed or separated), and chronic illnesses added statistically significantly to the prediction of substance use. Our findings showed that women aged 50 years and older used fewer substances than men in the same age group. However, widowed or separated individuals and those dealing with chronic illnesses were more likely to engage in substance use. These findings align with prior studies, which showed that more than half of older adults (ranging from 54.5% to 57.8%) engaged in substance use, with higher rates observed among those aged 65 years and older [26,27]. However, substance usage among older women and men varies based on the particular types of substances. Older women are more likely to use sedatives, weight loss medications, and pain relievers, while older men are more likely to consume alcohol [24,28,29]. In terms of chronic illnesses, Ahuja et al [11] found similar results to our findings, indicating a correlation between chronic illnesses and various substance use among the older adults. Specifically, this earlier work highlighted that reporting 2 or more chronic illnesses is linked to a higher probability of substance use, such as marijuana [11]. Regarding marital status, our results correspond with those of Johar et al [30]. Their research suggests that the experience of being widowed or separated is frequently tied to emotions of loneliness, social isolation, or lifestyle changes, which could impact substance use behavior [30]. Based on our findings, it is unclear whether the specific substances used among older women present an opportunity to delve into why substance use is relatively low in this group. Moreover, taking into account specific conditions such as dementia, arthritis, and high cholesterol may influence substance use to alleviate chronic illnesses; therefore, screening and assessment are important to identify the factors affecting substance use.

### Chronic Illness, COVID-19 Concerns, and Adults Aged 50 Years and Older

Individuals with dementia experience a loss of cognitive functioning, including rationality, memory, and logical thinking. This condition is recognized as one of the significant causes of dependency among adults aged 50 years and older [31]. The COVID-19 pandemic has raised concerns for individuals with dementia, who are at high risk of severe COVID-19 infection [32]. Those with dementia may face challenges in following safeguarding procedures, such as maintaining social distancing, wearing masks, and practicing hand hygiene, which all leave them more vulnerable to COVID-19 infection [33] and encounter difficulty communicating their health concerns and face barriers to accessing health care services during the COVID-19 pandemic [31]. The impact of the COVID-19 pandemic on adults aged 50 years and older with dementia has been reported, including worsening mental health while receiving live-in home health care [34] and poor health outcomes from COVID-19 hospitalization [35,36]. Individuals with arthritis may have significant concerns as they are at risk of COVID-19 infection. A recent study found that individuals with arthritis have a significantly higher risk of contracting COVID-19 infection [37]. This increased risk may be attributed to factors such as receiving treatment involving immunosuppressant medications, which can predispose them to COVID-19 infection and severe illness, potentially leading to hospitalization or death when compared with those with no arthritis [37,38]. In addition, the literature indicates that individuals with arthritis face an elevated risk of experiencing severe COVID-19 symptoms, particularly those associated with interstitial lung disease [39]. These findings may heighten concerns among individuals with arthritis about navigating the COVID-19 pandemic. Social isolation stemming from the COVID-19 pandemic poses a significant concern for individuals’ lifestyles, particularly those with high cholesterol. Managing high cholesterol necessitates lifestyle modifications such as adopting a healthy diet, engaging in regular physical activity, and reducing stress to mitigate cholesterol levels and delay-associated mortality [40]. However, adhering to these recommendations has been challenging during the pandemic, with individuals facing limited access to healthy food options, disrupted eating patterns, reduced physical activity, heightened stress levels, and compromised sleep quality [41]. These factors can impede efforts to control cholesterol levels and elevate the risk of progression to comorbidities such as metabolic syndrome and cardiovascular disease [42]. Furthermore, research indicates a correlation between cholesterol levels, higher body mass index, and susceptibility to COVID-19 infection, as well as the severity of COVID-19 illness [43-45]. These findings underscore the heightened concerns surrounding COVID-19 infection for individuals managing high cholesterol.

### The Impact of COVID-19 Pandemic on Employment in Adults Aged 50 Years and Older With Chronic Illness

Our findings indicate the effect of the COVID-19 pandemic on work among individuals with hypertension, lung disease, heart condition, stroke, or arthritis. According to the CDC, individuals with chronic illnesses are at higher risk for poor health outcomes and developing more serious complications from COVID-19 infection [46]. The Occupational Safety and Health Administration launched general guidance for employees and employers based on the CDC’s statement on recognizing personal health risk factors and helping facilitate their work [47]. For example, people with chronic illnesses are allowed to work from home every day to reduce contact with other coworkers. Moreover, employees with chronic illnesses reported higher levels of COVID-19 fear than employees with no chronic illnesses, which may affect how they work [48]. However, our findings indicate that the effect of the COVID-19 pandemic on work is not correlated with individuals with diabetes, depression, dementia, or high cholesterol, despite being listed in the CDC’s statement. These findings remain inconclusive in previous studies. Regarding the association between the effect of the COVID-19 pandemic and leaving the workforce entirely, our findings reported the significance of diabetes mellitus, while other chronic illnesses are not significant. Individuals with diabetes mellitus may have to maintain their financial stability in managing their diabetes treatment, especially oral medications and insulin regimens. On the other hand, other chronic illnesses may have a lower cost of health care than diabetes mellitus. A study conducted in Tanzania reported that the financial burden relative to household income in individuals with diabetes mellitus increased by 32.1% [49]. As a result, individuals with diabetes mellitus may have to continue working during the COVID-19 pandemic.

### Chronic Illness as a Mediator in the Relationship Between COVID-19 Concerns and Substance Use

Based on our analysis, chronic illnesses act as a mediator in the relationship between COVID-19 concerns and substance use. There are 2 main postulations drawn from the results. First, individuals with chronic illnesses face a higher risk of COVID-19 infection, leading to increased stress and anxiety, which may prompt them to use substances as a coping strategy. This could be due to concerns about visiting hospitals amid the pandemic, driving them toward substance use to manage their heightened emotions, fears, and health conditions. Second, individuals may turn to substances as a means of self-medication to alleviate the psychological distress caused by both their chronic illnesses and the added anxiety from the pandemic. However, it is important to note that the cross-sectional nature of the study limits our ability to establish the temporal relationship conclusively. Nevertheless, evidence suggests an uptick in substance use among adults aged 50 years and older with chronic conditions during the COVID-19 period. Studies, such as one conducted in Chicago, observed a surge in alcohol consumption among adults aged 50 years and older with chronic conditions at the onset of the pandemic, which then declined by late summer 2021 with the expiration of stay-at-home orders [50]. This indicates a possible impact of lockdown measures on substance use [50]. This effect could be especially pronounced among adults aged 50 years and older with chronic illnesses who were significantly affected by lockdown policies and harbored heightened concerns about the pandemic, potentially leading them toward substance use. Moreover, a network analysis involving 3075 adults from the United States and Canada found strong links among COVID-19 stress syndrome, COVID-19 disregard syndrome, and substance use [51]. Symptoms of traumatic stress and disregard for social distancing were particularly associated with alcohol and drug abuse [51]. Interestingly, concerns about the severity of the COVID-19 pandemic emerged as a central factor in the network, suggesting that targeting this concern could help reduce substance abuse [51]. Another study also proposed a pathway linking COVID-19 concerns to substance use among underserved populations such as patients with chronic illness [52]. It suggested that COVID-19 concerns and social isolation could impact health care access and worsen economic statuses, potentially driving individuals toward self-medication with substances such as opiates [52]. The relationship among COVID-19 concerns, chronic illnesses, and substance use is undoubtedly complex. However, it provides valuable insights and a framework for future efforts to mitigate substance use.

Moving forward, proactive measures are crucial to support adults with chronic conditions during pandemics. By addressing pandemic-related stress and improving health care access, we can help prevent reliance on substance use as a coping mechanism. This proactive approach is vital not only for individual well-being but also for the broader public health landscape. In addition, this call for health care providers to play a pivotal role in intensifying screening efforts for stress and substance use concerns, particularly among the older adults, may require enhanced training to address mental health issues effectively.

### Limitations

Several limitations are noteworthy. First, the exclusion of psychological illness, which is prevalent among older adults, might fail to fully grasp the correlation between psychological illness and substance use. Hence, future studies should incorporate psychological illness to foster a more comprehensive understanding of the subject. Second, self-reported substance use may underestimate actual prevalence due to social desirability bias. Therefore, future studies should integrate self-reported data with objective measures such as prescription records or biological markers to enhance accuracy in estimating substance use among older adults. Third, using a cross-sectional study design might fail to establish the relationship between COVID-19 concern and substance use. Therefore, future studies should consider a longitudinal approach to track substance use patterns among older adults over time, facilitating a clearer understanding of the temporal relationship between pandemic-related concerns and substance use. Finally, there is a modest *R*^2^ change between model 1 (adjusted for age, sex, race or ethnicity, number of years in school, and marital status) and model 2 (adjusted for sex and race or ethnicity) of 2 associations: (1) the association between characteristics of adults aged 50 years and older on substance use and (2) the association of chronic illness in adults aged 50 years and older and COVID-19 concern. Therefore, the findings should be interpreted with caution and future studies should include a larger sample to strengthen statistical analysis and result findings.

### Conclusions

Our study highlights the intricate relationship between chronic illnesses, COVID-19 infection, and substance use among adults aged 50 years and older. We found that age, sex, marital status, and chronic illnesses significantly influence substance use behaviors in this demographic. Older adults with certain chronic conditions exhibited heightened COVID-19 concerns, which, in turn, mediated their substance use. These findings underscore the importance of proactive support for older adults with chronic conditions during pandemics and the need for further research to elucidate these complex dynamics.

## Appendix

Multimedia Appendix 1 STROBE (Strengthening the Reporting of Observational Studies in Epidemiology) statement—checklist of items that should be included in reports of observational studies.

Multimedia Appendix 2 Association between chronic illness and COVID-19 Self-report and COVID-19 Test-based.

## Abbreviations

CDC: Centers for Disease Control and Prevention
HRS: Health and Retirement Study
IRB: institutional review board
OR: odds ratio
STROBE: Strengthening the Reporting of Observational Studies in Epidemiology
