# Supplementary material for: Exploring the Linkages Among Chronic Illness, Substance Use, and COVID-19 Infection in Adults Aged 50 Years and Older: Retrospective Cross-Sectional Analysis of National Representative Data
Source: JMIR Aging. 2024 Oct 15;7:e63024. doi: 10.2196/63024 (PMC11522661; doi:10.2196/63024)
Supplement: Multimedia Appendix 2 [file aging_v7i1e63024_app2.docx]

**Table S1.** The association between chronic illness of adult and older adults and COVID-19 self-report.

| **Chronic Illness** | | **B** | | **S.E.** | | **Sig.** | | **OR** | | **95% C.I. for OR** | | | |
| --- | --- | --- | --- | --- | --- | --- | --- | --- | --- | --- | --- | --- | --- |
|  |  |  |  |  |  |  |  |  |  | **Lower** | | **Upper** | |
| **Hypertension** | .455 | | .362 | | .209 | | 1.577 | | .775 | | 3.207 | |  |
| **Diabetes Meletus** | .490 | | .425 | | .248 | | 1.632 | | .710 | | 3.752 | |  |
| **Lung’s Disease** | .600 | | .631 | | .342 | | 1.822 | | .529 | | 6.277 | |  |
| **Heart Condition** | -.370 | | .389 | | .341 | | .691 | | .322 | | 1.480 | |  |
| **Stroke** | .414 | | .755 | | .583 | | 1.513 | | .345 | | 6.644 | |  |
| **Depression** | -.555 | | .358 | | .121 | | .574 | | .285 | | 1.157 | |  |
| **Arthritis** | -.122 | | .360 | | .736 | | .885 | | .437 | | 1.794 | |  |
| **High Cholesterol** | -.673 | | .350 | | .055 | | .510 | | .257 | | 1.013 | |  |

**Note.** COVID-19 self-reporting is a dependent outcome variable. The model was adjusted for sex and race/ethnicity.

Statistically significant at p-value < 0.05.

P = p-value, OR = Odds Ratio, CI = Confident Interval.

**Table S2.** The association between chronic illness of adult and older adults and COVID-19 test-based.

| **Chronic Illness** | **B** | **S.E.** | **Sig.** | **OR** | **95% C.I. for OR** | |
| --- | --- | --- | --- | --- | --- | --- |
|  |  |  |  |  | **Lower** | **Upper** |
| **Hypertension** | .579 | .487 | .235 | 1.785 | .686 | 4.639 |
| **Diabetes Meletus** | -.037 | .515 | .943 | .964 | .351 | 2.646 |
| **Lung’s Disease** | .214 | .698 | .759 | 1.239 | .315 | 4.869 |
| **Heart Condition** | -.456 | .474 | .336 | .634 | .250 | 1.606 |
| **Stroke** | -.720 | .722 | .319 | .487 | .118 | 2.004 |
| **Depression** | -.397 | .458 | .386 | .673 | .274 | 1.649 |
| **Arthritis** | .490 | .473 | .301 | 1.632 | .646 | 4.123 |
| **High Cholesterol** | -.412 | .462 | .373 | .662 | .268 | 1.639 |

**Note.** COVID-19 test-based is a dependent outcome variable. The model was adjusted for sex and race/ethnicity.

Statistically significant at p-value < 0.05.

P = p-value, OR = Odds Ratio, CI = Confident Interval.
